# Association of a second-trimester alpha-fetoprotein-to-transaminase ratio with preeclampsia: an exploratory retrospective cohort study

**DOI:** 10.1186/s12884-026-09876-7

**Published:** 2026-09-11

**Authors:** Murat Haksever, Uğurcan Zorlu, Sadık Orhun Aktaş, Hakkı Şerbetci, Şükrü Bakırcı, Dilek Şahin

**Affiliations:** Department of Perinatology, Ankara Bilkent City Hospital, University of Health Sciences, Ankara, Türkiye

**Keywords:** Preeclampsia, Alpha-fetoprotein, AST, ALT, Biomarker, Retrospective cohort

## Abstract

**Background:**

Maternal serum alpha-fetoprotein (AFP) is related to placental dysfunction, whereas aminotransferases are routinely available biochemical measures. We evaluated an empirically constructed AFP/(AST×ALT) index measured during second-trimester screening as an exploratory marker associated with subsequent preeclampsia. We specifically examined whether the index showed a consistent severity gradient and whether it provided clinically meaningful discrimination.

**Methods:**

This retrospective cohort study included 164 singleton pregnancies followed at a tertiary referral center between January 2022 and June 2024: 81 pregnancies without preeclampsia, 42 with preeclampsia without severe features, and 41 with preeclampsia with severe features. AFP, aspartate aminotransferase (AST), and alanine aminotransferase (ALT) measured at 16–18 weeks were retrieved. The AFP/(AST×ALT) index was calculated. Group comparisons were performed using variance-robust methods with post-hoc pairwise testing. Candidate diagnostic performance was explored using receiver-operating-characteristic analysis, and logistic regression was scaled per 0.1-unit increase in the index. Multivariable models included maternal age, body mass index, parity, and gestational age at sampling.

**Results:**

The index showed a non-monotonic pattern across groups: 0.200 ± 0.188 in pregnancies without preeclampsia, 0.801 ± 0.422 in preeclampsia without severe features, and 0.229 ± 0.239 in preeclampsia with severe features. The comparison between pregnancies without preeclampsia and preeclampsia with severe features was not significant, whereas both comparisons involving preeclampsia without severe features were significant. This pattern was driven largely by lower second-trimester AST and ALT values in the group without severe features. For preeclampsia presence, ROC analysis yielded an AUC of 0.738 (95% CI 0.654–0.812). A cutoff of 0.328 provided 61.4% sensitivity and 86.4% specificity. In multivariable analysis adjusted for maternal age, BMI, parity, and gestational age at sampling, the adjusted OR was 1.46 (95% CI 1.25–1.71) per 0.1-unit increase in the index.

**Conclusions:**

AFP/(AST×ALT) demonstrated an association with preeclampsia in this retrospective cohort, but the relationship was non-monotonic across clinical phenotypes and appeared strongly influenced by low transaminase values in the group without severe features. The index should therefore be regarded as exploratory rather than as a validated severity biomarker or stand-alone clinical test. Independent prospective validation is required.

## Background

Preeclampsia is a heterogeneous hypertensive disorder of pregnancy characterized by new-onset hypertension after 20 weeks of gestation together with proteinuria and/or maternal organ or uteroplacental dysfunction. Abnormal placentation, uteroplacental malperfusion, endothelial dysfunction, and an imbalance in angiogenic signaling are central to its pathophysiology [1–3]. Contemporary diagnostic frameworks are summarized in ACOG and ISSHP guidance [4, 5].

Maternal serum AFP is routinely available from second-trimester screening and has been associated with placentally mediated complications, including preeclampsia, fetal growth restriction, and preterm birth [6–8]. By contrast, AST and ALT are markers of hepatocellular injury and may become elevated when maternal end-organ involvement develops in established preeclampsia. Importantly, AST and ALT measured at 16–18 weeks precede the clinical diagnosis in most affected pregnancies and should therefore not be interpreted as measurements of preeclampsia-related hepatic injury.

The AFP/(AST×ALT) index examined in this study was an empirically constructed composite of routinely available analytes. Because multiplication of two correlated enzymes in the denominator can magnify small denominator values, the index has no established mechanistic or physical-unit interpretation and requires cautious evaluation. We therefore treated the present analysis as exploratory and aimed to determine (1) whether the index was associated with subsequent preeclampsia, (2) whether any association showed a consistent gradient across preeclampsia phenotypes, and (3) its preliminary discriminative performance.

## Methods

### Study design and participants

This retrospective cohort study was conducted at the Department of Perinatology, Ankara Bilkent City Hospital, a tertiary referral center, between January 2022 and June 2024. The final cohort comprised 164 pregnancies: 81 without preeclampsia, 42 with preeclampsia without severe features, and 41 with preeclampsia with severe features. The study is reported in accordance with the STROBE recommendations for observational studies [9].

Women aged 18–45 years with singleton pregnancies and available AFP, AST, and ALT results obtained during routine second-trimester screening at 16–18 weeks were eligible. Exclusion criteria were multiple gestation, chronic hypertension, pregestational diabetes mellitus, renal or autoimmune disease, known gastrointestinal or hepatobiliary disease, abnormal baseline liver-function tests, fetal structural anomalies or neural-tube defects, maternal or fetal mass lesions, and active smoking.

### Definitions of preeclampsia and severe features

Preeclampsia was defined according to contemporary ACOG/ISSHP criteria as new-onset systolic blood pressure ≥ 140 mmHg and/or diastolic blood pressure ≥ 90 mmHg on two occasions after 20 weeks in a previously normotensive woman, accompanied by proteinuria or, in the absence of proteinuria, maternal organ dysfunction and/or uteroplacental dysfunction. Severe features included persistent blood pressure ≥ 160/110 mmHg, thrombocytopenia, impaired liver function, renal insufficiency, pulmonary edema, or new-onset cerebral or visual symptoms, according to applicable criteria [4, 5]. The terms “preeclampsia without severe features” and “preeclampsia with severe features” are used throughout the revision rather than “mild” and “severe” preeclampsia.

Gestational age at preeclampsia diagnosis was not sufficiently complete in the retrospective records to permit a reliable early-onset (< 34 + 0 weeks) versus late-onset (≥ 34 + 0 weeks) subgroup analysis; this was therefore not used for inferential analysis.

### Laboratory measurements and index construction

AFP, AST, and ALT results were retrieved from the hospital laboratory information system. Measurements were performed in the hospital central laboratory using automated Siemens Healthineers analytical platforms. AFP was measured by automated immunoassay, whereas AST and ALT were measured using standardized automated enzymatic methods. Calibration and internal quality-control procedures were performed in accordance with the laboratory’s routine standard operating procedures and the manufacturer’s instructions.

The exploratory index was calculated as AFP/(AST×ALT), with AFP in the laboratory-reported concentration unit and AST/ALT in U/L. Because this mathematical construction has no established physical-unit interpretation and can be disproportionately affected by small denominator values, its distribution and underlying analytes were evaluated separately. The index was not considered a validated mechanistic measure of combined placental and hepatic injury.

### Outcomes

Neonatal outcomes evaluated individually included 1-minute Apgar score, 5-minute Apgar score, birth weight, gestational age at delivery, and neonatal intensive care unit (NICU) admission. Because the previously used composite “adverse outcome” combined heterogeneous events and was not sufficiently informative for clinical interpretation, it was not retained as an outcome in the revised analysis.

### Statistical analysis

Continuous variables were assessed for distributional shape using graphical inspection and Shapiro–Wilk testing. Because the AFP/(AST×ALT) index was right-skewed and variances differed across groups, both mean ± SD and median (IQR) were considered, and three-group comparisons were performed using Welch’s ANOVA with Games–Howell post-hoc testing. Categorical variables were compared using χ² or Fisher’s exact tests, as appropriate. Gestational age at sampling was explicitly compared across groups.

For exploratory discrimination of preeclampsia presence, ROC analysis was performed and AUC with 95% confidence interval was calculated. The Youden index was used to identify an exploratory cutoff, with sensitivity, specificity, positive predictive value, and negative predictive value reported. Because predictive values depend on disease prevalence and this single-center retrospective sample may not represent population prevalence, PPV and NPV were interpreted cautiously.

Logistic regression was reported per 0.1-unit increase in AFP/(AST×ALT), rather than per one-unit increase. The multivariable model included maternal age, BMI, parity, and gestational age at sampling. The primary inferential focus was preeclampsia presence; analyses according to severe features were considered exploratory because the index did not demonstrate a monotonic severity gradient. Multiple secondary comparisons were interpreted as hypothesis-generating.

All statistical analyses were performed using SPSS version 25.0 (IBM Corp., Armonk, NY, USA). A two-sided p value < 0.05 was considered statistically significant.

### Ethics

The study was approved by the Ethics Committee for Medical Research at Ankara City Hospital (TABED 2-24-538) and was conducted in accordance with the Declaration of Helsinki. Owing to the retrospective design, the requirement for informed consent was waived by the ethics committee.

## Results

The cohort included 164 pregnancies: 81 without preeclampsia, 42 with preeclampsia without severe features, and 41 with preeclampsia with severe features. Maternal age, gravidity, parity, BMI, and gestational age at second-trimester sampling did not differ significantly among groups (Table 1).

**Table 1:** Baseline maternal characteristics and gestational age at second-trimester sampling according to study group

Variable	No PE (*n* = 81)	PE without severe features (*n* = 42)	PE with severe features (*n* = 41)	*p* value*
Age, years	30.92 ± 6.32	29.72 ± 6.84	29.68 ± 5.77	0.465
BMI, kg/m²	25.89 ± 4.21	26.91 ± 4.25	27.20 ± 3.58	0.169
Gravida	2.12 ± 1.22	1.98 ± 1.02	1.95 ± 0.92	0.645
Parity	1.23 ± 1.03	0.98 ± 0.90	0.98 ± 0.88	0.235
Gestational age at sampling, weeks	17.09 ± 0.51	17.04 ± 0.43	17.07 ± 0.55	0.831

*Welch ANOVA was used for continuous variables

AFP/(AST×ALT) differed significantly across groups, but the pattern was non-monotonic. Mean values were 0.200 ± 0.188 in pregnancies without preeclampsia, 0.801 ± 0.422 in preeclampsia without severe features, and 0.229 ± 0.239 in preeclampsia with severe features. Games–Howell post-hoc testing showed significant differences between pregnancies without preeclampsia and preeclampsia without severe features (*p* < 0.001), and between preeclampsia without and with severe features (*p* < 0.001), whereas pregnancies without preeclampsia and those with severe features did not differ significantly (*p* = 0.772) (Table 2).

**Table 2:** Second-trimester AFP, AST, ALT, and AFP/(AST×ALT) values across study groups with pairwise post-hoc comparisons

Analyte	No PE	PE no severe features	PE severe features	Overall *p*	No PE vs. no severe	No PE vs. severe	No severe vs. severe
AFP	35.220 ± 14.540	42.570 ± 12.280	32.260 ± 13.270	0.001	0.011	0.500	0.001
AST (U/L)	16.720 ± 7.620	7.860 ± 5.470	14.780 ± 5.800	< 0.001	< 0.001	0.266	< 0.001
ALT (U/L)	18.890 ± 15.500	10.020 ± 5.760	16.610 ± 10.510	< 0.001	< 0.001	0.605	0.002
AFP/(AST×ALT)	0.200 ± 0.187	0.804 ± 0.421	0.229 ± 0.239	< 0.001	< 0.001	0.772	< 0.001

Overall comparisons used Welch ANOVA; pairwise p values were obtained using Games–Howell post-hoc tests

*AFP* alpha-fetoprotein, *AST* aspartate aminotransferase, *ALT* alanine aminotransferase, *PE* preeclampsia

The underlying analytes clarified this pattern. AFP was moderately higher in preeclampsia without severe features, whereas AST and ALT were markedly lower in that group. Pairwise comparisons between pregnancies without preeclampsia and preeclampsia with severe features were not significant for AFP, AST, ALT, or the composite index (Table 2). These findings indicate that the marked elevation of AFP/(AST×ALT) in preeclampsia without severe features was substantially influenced by the denominator and should not be interpreted as a progressive severity response.

The AFP/(AST×ALT) distribution was right-skewed. Median (IQR) values were 0.154 (0.091–0.233) in pregnancies without preeclampsia, 0.689 (0.535–0.899) in preeclampsia without severe features, and 0.147 (0.088–0.328) in preeclampsia with severe features (Table 3).

**Table 3:** Distribution of the AFP/(AST×ALT) index according to study group

Group	Median	IQR	Min–max
No PE	0.154	0.091–0.233	0.028–1.190
PE without severe features	0.689	0.535–0.899	0.265–2.133
PE with severe features	0.147	0.088–0.328	0.020–1.321

*IQR* interquartile range

For the exploratory outcome of preeclampsia presence, ROC analysis yielded an AUC of 0.738 (95% CI 0.654–0.812). A Youden-derived cutoff of 0.328 yielded 61.4% sensitivity and 86.4% specificity, with a PPV of 82.3% and NPV of 68.6% in this cohort. In multivariable logistic regression adjusted for maternal age, BMI, parity, and gestational age at sampling, AFP/(AST×ALT) remained associated with preeclampsia presence (adjusted OR 1.46, 95% CI 1.25–1.71 per 0.1-unit increase) (Table 4).

**Table 4:** Exploratory diagnostic performance and adjusted association of AFP/(AST×ALT) with the presence of preeclampsia

Metric	Estimate
AUC	0.738 (bootstrap 95% CI 0.654–0.812)
Youden cutoff	0.328
Sensitivity	61.4%
Specificity	86.4%
PPV	82.3%
NPV	68.6%
Adjusted OR per 0.1-unit increase	1.46 (95% CI 1.25–1.71)
Adjusted covariates	Age, BMI, parity, gestational age at sampling

*AUC* area under the receiver operating characteristic curve, *PPV* positive predictive value, *NPV* negative predictive value, *OR* odds ratio, *CI* confidence interval

The non-monotonic three-group pattern precludes interpretation of the ratio as a general severity gradient. Accordingly, ROC and regression findings were interpreted as measures of association and within-cohort discrimination for preeclampsia presence rather than as evidence of a validated clinical screening test.

## Discussion

The principal finding of this study is not a monotonic severity relationship but a non-linear phenotype-specific pattern. AFP/(AST×ALT) was markedly elevated in the group that later developed preeclampsia without severe features, whereas values in preeclampsia with severe features were close to those of pregnancies without preeclampsia. This distinction is central: an omnibus group difference does not establish that the index increases with disease severity.

The pattern is largely explained by the denominator. AFP was moderately higher in the group without severe features, consistent with literature linking elevated maternal AFP to placental dysfunction [6–8, 10, 11]. In contrast, second-trimester AST and ALT were substantially lower in that group. Because both enzymes are multiplied in the denominator, simultaneous reductions can cause a disproportionate increase in the ratio. Low second-trimester transaminase values are not an established preclinical feature of preeclampsia; therefore, the observed enzyme pattern may reflect biological heterogeneity, residual confounding, or sampling variability rather than an early hepatic manifestation of the disease.

This consideration also changes the biological interpretation of the index. AST and ALT measured at 16–18 weeks should not be described as indicators of later preeclampsia-related hepatic injury. Hepatic involvement is a feature of established maternal disease and severe features, whereas the measurements used here were obtained before clinical onset. Accordingly, we interpret AFP as a potentially placental-associated analyte and early AST/ALT as baseline biochemical covariates rather than as a contemporaneous hepatic component of preeclampsia. Importantly, neurological complications such as posterior reversible encephalopathy syndrome (PRES) may accompany hypertensive disorders of pregnancy and HELLP syndrome, and case-based evidence shows that neurological manifestations can occasionally precede full biochemical deterioration; PRES has also been described in other acute hypertensive and endothelial-injury settings [12, 13]. These observations further caution against assuming that a single early biochemical measure necessarily parallels the later clinical expression of end-organ disease.

The multiplicative construction AFP/(AST×ALT) was exploratory and has no established physiological unit or validated mechanistic model. Multiplying two correlated aminotransferases also increases sensitivity to small denominator values. We therefore report the underlying analytes, pairwise contrasts, and distribution of the index and explicitly avoid presenting the formula as a validated pathophysiological composite. Alternative formulations, such as modeling AFP, AST, and ALT separately or using standardized multivariable combinations, should be investigated in independent data before any particular formula is preferred. This cautious approach is consistent with the broader experience of exploratory composite-ratio biomarkers in preeclampsia [14].

The index showed moderate discrimination for preeclampsia presence but did not demonstrate a general severity gradient. An AUC of 0.738 indicates statistical discrimination within this cohort, but does not by itself establish clinical usefulness. Established risk algorithms incorporate maternal characteristics, blood pressure, uterine-artery Doppler, and/or angiogenic biomarkers; the sFlt-1/PlGF ratio, for example, has been prospectively evaluated in women with suspected preeclampsia [15, 16]. The incremental value of AFP/(AST×ALT) beyond established approaches remains unknown.

Neonatal outcomes were evaluated individually rather than as a heterogeneous composite. This approach was chosen because perinatal outcomes are influenced by gestational age at disease onset and delivery, fetal growth, management decisions, and severity of maternal disease, and combining clinically distinct events may obscure outcome-specific associations. Recent Doppler evidence also indicates that maternal physiological exposures can be accompanied by subclinical alterations in fetal cardiac function and hemodynamics even when conventional pregnancy outcomes are not the primary signal, supporting assessment of fetal effects beyond crude neonatal endpoints alone [17].

This study has several limitations. It is retrospective and single-center, with a modest sample size and particularly limited subgroup event counts. Residual confounding remains possible because previous preeclampsia, assisted conception, medication exposure, nutritional status, and other unmeasured factors may influence the observed biochemical pattern. The proposed index was exploratory and was not externally validated. Multiple secondary analyses raise the possibility of chance findings. There were no longitudinal biomarker measurements, gestational age at preeclampsia diagnosis was not sufficiently complete for reliable early- versus late-onset subgroup analysis, and the index was not compared directly with validated prediction models or angiogenic biomarkers such as sFlt-1/PlGF. The non-monotonic group pattern and the unusually low AST/ALT values in the group without severe features are particularly important limitations and require replication.

Strengths include the use of routinely available analytes obtained within a narrow second-trimester window, explicit examination of the component biomarkers rather than the composite alone, variance-robust pairwise comparisons, clinically interpretable effect scaling, adjustment for key measured covariates, and quantification of within-cohort diagnostic performance.

## Conclusions

The second-trimester AFP/(AST×ALT) index was associated with preeclampsia in this retrospective cohort, but the relationship was non-monotonic across preeclampsia phenotypes and was strongly influenced by low AST and ALT values in the group without severe features. The index should not be interpreted as a validated marker of disease severity or as a stand-alone clinical test. Prospective external validation and direct comparison with established clinical and angiogenic prediction strategies are required before clinical application.

## Data Availability

The datasets used and/or analyzed during the current study are available from the corresponding author on reasonable request, subject to institutional and ethical regulations.
